# Thermoreversible Gel-Loaded Amphotericin B for the Treatment of Dermal and Vaginal Candidiasis

**DOI:** 10.3390/pharmaceutics11070312

**Published:** 2019-07-03

**Authors:** Lilian Sosa, Ana Cristina Calpena, Marcelle Silva-Abreu, Lupe Carolina Espinoza, María Rincón, Nuria Bozal, Oscar Domenech, María José Rodríguez-Lagunas, Beatriz Clares

**Affiliations:** 1 Department of Pharmacy and Pharmaceutical Technology and Physical Chemistry, School of Pharmacy and Food Sciences, University of Barcelona, 08028 Barcelona, Spain; 2 Institute de Nanoscience and Nanotechnology (IN2UB), University of Barcelona, 08028 Barcelona, Spain; 3 Departamento de Química y Ciencias Exactas, Universidad Técnica Particular de Loja, Loja 1101608, Ecuador; 4 Department of Biology, Healthcare and the Environment, School of Pharmacy and Food Sciences, University of Barcelona, 08028 Barcelona, Spain; 5 Department of Biochemistry and Physiology, Faculty of Pharmacy and Food Sciences, University of Barcelona, 08028 Barcelona, Spain; 6 Institut de Recerca en Nutrició i Seguretat Alimentària (INSA), Universitat de Barcelona, 08921 Barcelona, Spain; 7 Department of Pharmacy and Pharmaceutical Technology, School of Pharmacy, University of Granada, 18071 Granada, Spain

**Keywords:** thermoreversible gel, poloxamer 407, candidiasis, amphotericin B, skin and vaginal mucosa

## Abstract

The present study was designed to develop a thermoreversible gel of Pluronic (P407) loaded amphotericin B (AmB-gel) for the dermal and vaginal treatment of candidiasis. P407 was used as a copolymer to exploit potential advantages related to increasing drug concentration in the tissue layer in order to provide a local effect. Parameters including internal structure, swelling, porosity, and short-term stability were determined. In addition, drug release profile and ex vivo skin and vaginal permeation studies were carried out. Antifungal efficacy was evaluated against strains of *Candida* spp. and atomic force microscopy (AFM) supported the results. The tolerance of AmB-gel was studied by evaluating biomechanical properties of skin and determining the irritation level in scarified rabbit skin supported by histological analysis. Results confirmed the development of a thermoreversible AmB-gel with high porosity exhibiting Newtonian behavior at 4 °C and pseudoplasticity at 32 °C as well as optimal stability for at least 90 days. The Amb-gel provided a sustained drug release following a Boltzmann sigmoidal model. Non permeation was observed in skin and vaginal mucosa, showing a high retained amount of AmB of 960.0 and 737.3 µg/g/cm^2^, respectively. In vitro antifungal efficacy showed that AmB-gel was more effective than Free-AmB in inhibiting strains of *Candida* spp. and these results were corroborated by AFM. Finally, tolerance studies showed that its application did not induce skin irritation nor alter its biophysical properties. Together, these results confirmed that AmB-gel could be proposed as a promising candidate for the clinical status in the treatment of skin and vaginal candidiasis.

## 1. Introduction

Cutaneous fungal infections are a significant cause of morbidity and constitute a critical health issue on a global scale. The most identified fungal pathogenesis that affects people worldwide is based on superficial skin, vaginal and nail infections, with approximately 1.7 billion individuals affected. This has led to an increase of superficial mycoses higher than 20% in recent decades, especially in patients who are immunocompromised or hospitalized [1,2] as well as in cases of topical burn wound infections [3]. Among these fungal infections, various species of Candida cause superficial complications known as candidiasis [4]. Categorically, *Candida albicans* is reported to be the fourth most frequent cause of infection [5].

The first-line drugs used for the treatment of these mycoses are the Azoles antifungal compounds such as fluconazole, ketoconazole, voriconazole, itraconazole, clotrimazole, among others [6,7]. However, mechanisms of resistance to these antifungals have been detected [8,9]. The appearance of these resistance mechanisms leads to the use of other antifungals such as amphotericin B (AmB), especially in immunosuppressed patients [10,11].

AmB is a polyene macrolide of a broad-spectrum with high activity against the most frequently occurring fungi involved in cutaneous mycoses, including Candida [12]. It is considered the gold standard in the treatment of fungal infections and has been found to be effective against azole-resistant fungi due to the creation of transmembrane channels by complexation with the membrane sterols which results in fungal cell death. However, it is not without side effects in systemic administration due to the self-assembly process into higher-order molecular forms [13]. From a physicochemical point of view, AmB is a poorly hydrosoluble, amphoteric, amphiphilic molecule and is difficult to solubilize in organic solvents. These properties block an optimal permeation of the drug through the stratum corneum (SC), which is the major barrier that limits permeation of external substances. For this reason, there is no topical formulation of AmB commercially available at the moment [14]. This fact evidences the need to develop new formulations using excipients with permeation-enhancing properties in order to facilitate the penetration of drug into SC and its distribution from SC to epidermis and dermis. Therefore, the research of new therapeutic approaches for the treatment of superficial mycoses, including dermal and vaginal administration from galenical perspective, is a challenging yet necessary undertaking.

The topical administration of antimicrobials for superficial mycoses offers several advantages, particularly for direct application at the site of infection. Among these are the prevention of systemic toxicity of the drug, effectiveness at an eminently local level and insurance that a sufficient amount of drug is retained in the skin [15].

With the aim of achieving an improved release of AmB, this drug was incorporated to Poloxamer 407 (P407), which is a copolymer of amphiphilic nature consisting of a central hydrophobic block of polypropylene oxide (PPO) flanked by hydrophilic polyethylene (PEG) blocks (PEG–PPO–PEG). The P407 prepared above >18% shows an aqueous solution (4–5 °C) that turns into a gel ~32 °C, making it an ideal candidate for thermoreversible delivery [16]. P407 is a low toxicity excipient approved by the Food and Drug Administration (FDA) [17]. Moreover, it has beneficial properties that help to promote and improve drug permeation through the skin and mucosa [18].

Therefore, an approach to deliver AmB formulated into P407 gel form specifically developed for skin and vaginal treatment against *Candida* spp. was developed. This work has accomplished detailed research of (i) development and physicochemical characterization of AmB-gel for topical administration, (ii) biopharmaceutical studies (release profile) and ex vivo permeation studies, (iii) in vitro antifungal activity and its effect on yeast cells by atomic force microscopy (AFM), and (iv) biomechanical properties and tolerance assay complemented with histological studies.

## 2. Materials and Methods

### 2.1. Materials

AmB was obtained from Acofarma (Barcelona, Spain). Pluronic^®®^ F127 (P407) was obtained from Fagron (Barcelona, Spain). Dimethyl sulfoxide (DMSO), methanol, castor oil and *N*,*N*-dimethylformamide were obtained from Sigma-Aldrich (Darmstadt, Germany). Transcutol^®®^ P and Propylene glycol were kindly provided by Gattefossé (Barcelona, Spain). The water used in all experiments was obtained from a Milli-Q^®®^ Plus System (Millipore Co., Burlington, MA, USA). All the chemicals and reagents were of analytical grade.

### 2.2. Preparation of AmB-Gel

Firstly, a selection of solvents was tested to determine the solubility of AmB. Transcutol^®®^ P, Propylene glycol, *N*,*N*-dimethyformamide, Castor oil and DMSO were analyzed. The DMSO was selected as the best solubilizing component of AmB. Shortly afterwards 25 g of P407 were dispersed on ultrapure water at 4 °C up to 100 mL. Subsequently, 60 mg of AmB were dissolved in 10 mL of DMSO, of which 1 mL was slowly added under stirring to 20 mL of P407 solution at 4 °C. The resulting solution was adjusted to pH 6.5 with NaOH 2N solution, thus obtaining an AmB-gel at a final concentration of 0.03%.

### 2.3. Physiochemical Characterization

To analyze the morphology, the AmB-gel was dried over a period of 8 days using a desiccator with provision for a vacuum. Once it was dried, a small quantity was coated with carbon as a conductor agent. The internal structure of the gel was examined by scanning electron microscopy (SEM) using a JEOL J-7100F (Peabody, Massachusetts, MA, USA).

#### 2.3.1. Swelling and Degradation Tests

The swelling ratio (SR) and degradation as percentage of weight loss (WL) were accomplished by a gravimetric method. Half a gram of dried AmB-gel or fresh AmB-gel was used to carry out swelling and degradation tests, respectively. In both experiments the sample was incubated in PBS (pH = 5.5) at 32 °C for 30 min. Samples (*n* = 3) were removed and weighed after blotting the surface water at predetermined time intervals of 5 min. The PBS uptake was carried out in triplicate.

The SR ratio was calculated using the following equation and expressed by kinetic modeling:

(1) $$\mathrm{SR}=\frac{W_{s}-W_{d}}{W_{d}}$$

where *W_s_* is the weight of the swollen AmB-gel at 5 min intervals and *W_d_* is the weight of dried gel.

WL was calculated following the equation and expressed by kinetic modeling.

(2) $$\mathrm{WL}\;\left(\%\right)=\;\frac{W_{i}-W_{d}}{W_{i}}\;\times 100$$

where *W_i_* is the initial weight of AmB-gel and *W_d_* the weight of gel at different times.

#### 2.3.2. Porosity Study

The porosity percentage (P) was calculated by displacement of the solvent. The method consisted of immersing the previously dried AmB-gel in absolute ethanol for 2 min and then weighing after the excess ethanol on the surface was blotted. The porosity percentage was calculated using the following equation.

(3) $$P=\;\frac{w2-w1}{\rho \times \;V}\;\times 100$$

where, *w1* represents the weight of the dried AmB-gel to be immersed in ethanol, *w2* represents the weight of AmB-gel after being immersed in ethanol, ρ is the density of absolute ethanol, and *V* is the volume of the gel.

### 2.4. Stability Study

The AmB-gel was stored at two temperatures: 4 °C and 25 °C. The pH values of formulation were measured in a Crison 501 digital pH/mV-meter (Crison Instruments, Barcelona, Spain) for a period of 90 days (1, 30, 60, and 90 days) at both temperatures. Values were reported as the mean ± standard deviation (SD) of six replicates.

For the chemical stability studies, the formulation was suspended in *N*,*N*–dimethylformamide:methanol:water (55:15:30, *v*/*v*/*v*) and the amount of drug was quantified by a previously validated method of High Performance Liquid Chromatography (HPLC) (Waters, Milford, MA, USA) [12].

The short-term stability of AmB-gel was studied for a period of 90 days at 4 °C and 25 °C. A TurbiScan Lab^®®^ (Formulaction Co., L’Union, France) was used to analyze the destabilization phenomena by transmission and light retrodispersion using a pulsed near-infrared light source (γ = 880 nm) at 25 °C over a span of 90 days.

### 2.5. Rheological Studies

The rheological measurements were performed using a Thermo Scientific Haake Rheostress 1 rotational rheometer (Thermo Fisher Scientific, Kalsruhe, Germany) equipped with cone plate geometry (60 mm diameter, 2° angle) with mobile upper cone Haake C60/2° Ti (0.105 mm gap). Viscosity and flow curves were rehearsed at 4 °C and 32 °C in triplicate. The shear rate ramp program included: 0→50 s^−1^ (3 min), 50 s^−1^ (1 min), and 50→0 s^−1^ (3 min). Obtained data were then fitted to different mathematical models: Newton, Bingham, Ostwald-de Waele, Cross, Casson, and Herschel–Bulkley.

### 2.6. Gelation Time and Spreadability Test

Ten milliliters of AmB-gel was added to a transparent vial with a magnetic bar and placed in a low temperature water bath. The solution was then heated to 37 ± 0.1 °C while being stirred (400 rpm). The gelation time was measured in triplicate once the magnetic bar stopped moving due to gelation.

The spreadability of AmB-gel was determined in triplicate as follows; 0.5 g of formulation was placed within a 1 cm diameter circle previously marked on a glass plate, after which a second glass plate was subsequently placed without sliding. A series of weights (15, 22, 27, 29, 32, and 37 g) were successively added and allowed to rest for 2 min each at 4 °C. The same operation was repeated at 32 °C but using different weights (100, 200, and 300 g). The diameters (cm) of the circle spreads were measured and recorded as comparative values. Experimental data were then fitted to mathematical models using GraphPad Prism^®®^ version 6.0 (GraphPad Software Inc., San Diego, CA, USA).

### 2.7. In Vitro Release and Kinetic Evaluation

In vitro release studies were performed in vertical diffusion Franz cells (FDC-400, Vidra-Foc, Barcelona, Spain) using nylon membranes.

DMSO solution continuously stirred at 32 ± 1 °C was used as the receptor medium to accomplish sink conditions. An aliquot of 1.3 mL of the AmB-gel was placed in the donor phase. Aliquots of 300 µL were collected from the receptor compartment at different times for 24 h and replaced with an equal volume of tempered DMSO kept under stirring at 600 rpm. AmB was quantified by previously validated HPLC [12]. Results are reported as the mean ± SD of six replicates.

Experimental data were fitted to five kinetic models (zero-order, first-order, Peppas–Korsmeyer, Higuchi, and Weibull function) by nonlinear least squares regression using GraphPad Prism^®®^ version 5.01. The best fit was selected based on the Akaike’s Information Criterion (AIC) and coefficient determination (*r^2^*).

### 2.8. Ex Vivo Permeation Studies: Skin and Vaginal Mucosa

Ex vivo permeation studies were performed as described in Section 2.7 using skin samples with thicknesses of 400 μm or Vaginal porcine mucosae as membranes. Transcutol P^®®^ was used as the receptor medium which was kept at 32 ± 0.5 °C (Human skin) and 37 ± 1 °C (Vaginal porcine mucosa). The experiment was performed with a diffusion area of 0.64 cm^2^. The human skin was obtained from the abdominal region of a healthy woman after abdominal plastic surgery. The experimental protocol was approved by the Bioethics Committee of Barcelona SCIAS Hospital (Barcelona, Spain) (ref: BEC/001/16) and written informed consent was provided by the volunteer. Integrity of skin was evaluated by measuring the transepidermal water loss (TEWL) with a DermaLab^®^ module (Cortex Technology, Hadsund, Denmark), exhibiting values below 10 g/m^2^‧h.

Vaginal porcine mucosae were obtained under veterinary supervision, from three- to four-month-old pigs after sacrifice using an overdose of sodium thiopental at the Animal Facility at Bellvitge Campus of Barcelona University (Barcelona, Spain) in accordance with protocols prescribed by the Animal Experimentation Ethics Committee of the University of Barcelona, Spain (CEEA-UB). Mucosa was placed into of Hank’s balanced salt solution (HBSS) and refrigerated until use. The experiments were carried out for 36 h (Human skin) and 6 h (Vaginal porcine mucosae), respectively.

At the end of the permeation study, the human skin and porcine vaginal mucosa were removed from the Franz diffusion cell, cleaned with a dodecyl sulfate solution 0.05% and washed in distilled water. AmB retained in the skin and vaginal mucosa was extracted with DMSO for a period of 20 min under cold sonication in an ultrasound bath. The resulting samples were measured by HPLC.

### 2.9. Antifungal Efficacy

The minimal inhibitory concentration (MIC), defined as the lowest concentration of an antimicrobial agent that inhibits the growth of a microorganism, was calculated by the broth microdilution method against *C. albicans* ATCC 10231, *C. glabrata* ATCC 66,032, and *C. parapsilosis* ATCC 22,019 strains (American Type Culture Collection, Manassas, VA, USA) following the procedure outlined by the European Committee for Antimicrobial Susceptibility Testing Guidelines (EUCAST) [19] and the Reference method CLSI M27-A3 [20].

This Standards Method provides a valid procedure for testing the susceptibility of glucose-fermenting yeasts to antifungal agents by determining the MIC. A synthetic medium containing RPMI-1640, glutamine, pH indicator without bicarbonate, and glucose 2% *w*/*v*: RPMI-1640 2% G (Invitrogen, Madrid, Spain) was used for working cultures. pH was adjusted to 7.0 with 1 M sodium hydroxide and the resulting solution was filtered using a 0.22 µm filter. As samples were not sterile, chloramphenicol was added to the RPMI medium at a final concentration of 500 µg/mL. The yeast strain was first cultured on Sabouraud Dextrose agar (Invitrogen, Madrid, Spain) at 30 °C for 48 h before testing. The inoculums were prepared by suspending colonies in sterile distilled water to achieve a density equivalent to 2 McFarland standards, the counting of which took place in a Neubauer Chamber shortly afterwards (1 to 5 × 10^6^ Colony Forming Unit, CFU/mL). Working suspension was prepared by diluting the standardized suspension in sterile distilled water (1:10) in order to prepare 1 to 5 × 10^5^ CFU/mL. The test was performed using 96-well polystyrene sterile microdilution plates. Serial dilutions from 75 to 0.009 μg/mL of the following forms were compared. (1) Free-AmB (AmB solution first dissolved in DMSO at 300 μg/mL and afterwards in RPMI-1640 double strength at 150 μg/mL). (2) AmB-gel (AmB gel dissolved in RPMI-1640 double strength at 150 μg/mL). (3) Blank-gel (Blank-gel dissolved in RPMI-1640 double strength at 12.5–0.00075%).

Finally, 100 μL of inoculum was added to all the wells. In addition, 3 posts were reserved in each plate for both the positive control (300 μL of the inoculum) and negative control (300 μL of the culture broth). The plates were read at t_0_ and then at 24 h and 48 h after incubation at 30 °C, with a microplate reader model 680 (Bio-Rad, Madrid, Spain) at λ = 620 nm.

### 2.10. Atomic Force Microscopy (AFM)

#### 2.10.1. Images

*C. albicans* cells were exposed to AMB-gel and Blank-gel during a period of 4 h. The cells were collected by centrifugation and washed three times with PBS solution (pH 7.40) in order to obtain a final suspension of 1 × 10^6^ yeast cells/mL. The suspension was filtered through SMWP01300 Millipore^®^ filters (Merck Chemical and Life Science, S.A, Madrid, Spain) with a pore size similar to the yeast size followed by gentle rinsing with PBS in order to clean the filter from non trapped cells, then subsequently cut and attached to a steel disk using a small piece of double-sided adhesive tape. Finally, the sample was transferred to the AFM liquid cell while avoiding dewetting. Throughout the procedure, MSNL-10, V shaped silicon nitride cantilevers (Bruker AFM Probes, Camarillo, CA, USA) with a nominal spring constant of 0.03 N/m were used.

Prior to measuring the nanomechanics the spring constants of the cantilevers were determined by thermal noise method. AFM images were acquired in contact mode by minimizing the force during the scan and continuously adjusting the set point with a 0° scan angle at a scan rate of 1.5 Hz. Images were processed using NanoScope^®®^ analysis software (Bruker, AXS Co., Madison, WI, USA).

#### 2.10.2. Force-Distance Curves

Mechanical properties were measured by recording arrays of 32 × 32 force curves, using a maximum force of 0.5–1 nN to avoid sample damage, a contact time of 100 ms, and approach and retract speeds of 1.0 µm/s. The Young’s modulus of the yeast cells was determined as a first approximation by using the Hertz model.

### 2.11. In Vivo Tolerance Study by Evaluating Biomechanical Properties of Human Skin

Ten female volunteers between 25 and 35 years old with healthy skin participated in the study. The study was approved by the CEEA-UB according to the recommendations outlined in the Declaration of Helsinki (ref: IRB00003099) and all volunteers provided written informed consent [21]. All participants were requested not to use skin care cosmetics on the test areas of application for two days prior to the study. Skin temperature, transepidermal water loss (TEWL), and stratum corneum hydration (SCH) were determined using a thermometer ST500, a Tewameter TM 300, and a Corneometer CM 825 (Courage-Khazaka electronic GmbH, Cologne, Germany), respectively [22]. The measurements of these parameters were made before applying the AmB-gel (basal readings), immediately after the application of 0.5 mL/cm^2^ (t_0_) and after 2 h of application on the flexor side of the left forearm.

### 2.12. In Vivo Tolerance Study by Draize Assay and Histological Analysis

The tolerance of the formulation was evaluated using scarified rabbit skin. The blank-gel and AmB-gel were tested on New Zealand albino male rabbits (2 kg) according to the guidelines provided by the CEEA-UB. The rabbits were acclimated over a 5-day period before the study and were classified into three groups (*n* = 3/group): Group A (Blank-gel), Group B (Skin scarified-control group), and Group C (AmB-gel). The surrounding area of the dorsal trunk was shaved with clippers where a square was drawn for scarification with a razor before beginning the assay. After 30 min, a volume of 0.5 mL of either Blank-gel or AmB-gel was topically applied on the scarified skin of each corresponding group while group B was not exposed to any treatment. This area was protected with gauze and secured with hypoallergenic sticking plaster for 48 h. The signal of edema and erythema were determined after 24 h and 48 h of exposure. Both scores were established according to the degree of severity and the primary irritation index value was calculated. The treatment was classified according the reported specifications: “nonirritant” (<0.5), “irritant” [2,3,4,5], or “highly irritant” [5,6,7,8,23]. Afterwards, the rabbits were anesthetized and euthanized with sodium pentobarbital.

For histological analysis, the samples of back skin from the rabbits were cut and set up for 24 h in 4% buffered formaldehyde at room temperature. After fixation, all samples were paraffin embedded in paraffin blocks, cut into 5 µm sections, and mounted on microscope slides. Afterwards, the samples were stained with hematoxylin and eosin and finally viewed on blind coded samples under a light microscope (Olympus BX41 and Olympus XC50 camera) with 100× magnification for the evaluation of the tissue structure.

### 2.13. Statistical Analysis

Obtained experimental data were analyzed by one-way analysis of variance (ANOVA). Comparisons of findings were done by multiple comparison test. A *p* value < 0.05 was established as an indicator of statistically significant differences (SSD).

## 3. Results

### 3.1. Physicochemical Characterization

**Figure 1:**
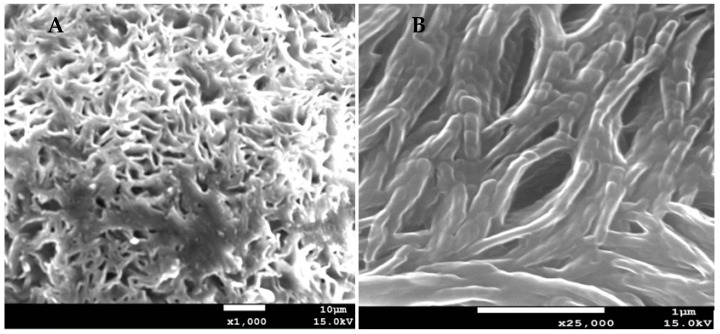
Morphological characterization by SEM. (**A**) AmB-gel porous sponge structure (×1000). (**B**) AmB-gel tubular appearance interconnected (×25,000).

Figure 1, shows SEM micrographs of AmB-gel, which exhibited a heterogeneous structure with the formation of interconnected capillary channels in the form of holes similar to a porous sponge.

**Figure 2:**
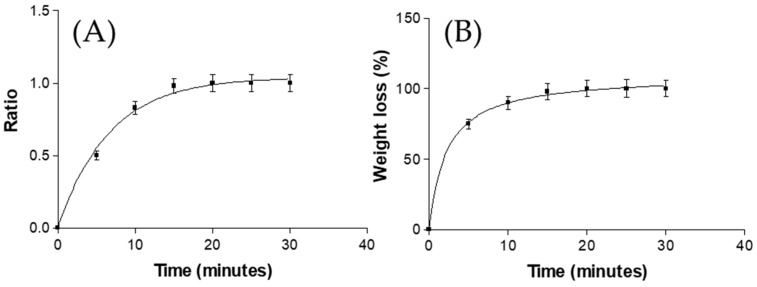
(**A**) Swelling ratio and (**B**) percentage of weight loss degradation of AmB-gel.

The swelling process of AmB-gel followed a first-order kinetic model, which was represented by the kinetic constants k = 0.26 min^−1^ (*r*^2^ = 0.9986) (Figure 2). The degradation process of AmB-gel was completed in 20 min and followed a hyperbola model with a kinetic constant of 0.15 min^−1^ (*r*^2^ = 0.9975), whereas the percentage of P of AmB-gel was ~82.01 ± 0.5%. 

### 3.2. Stability Study

**Figure 3:**
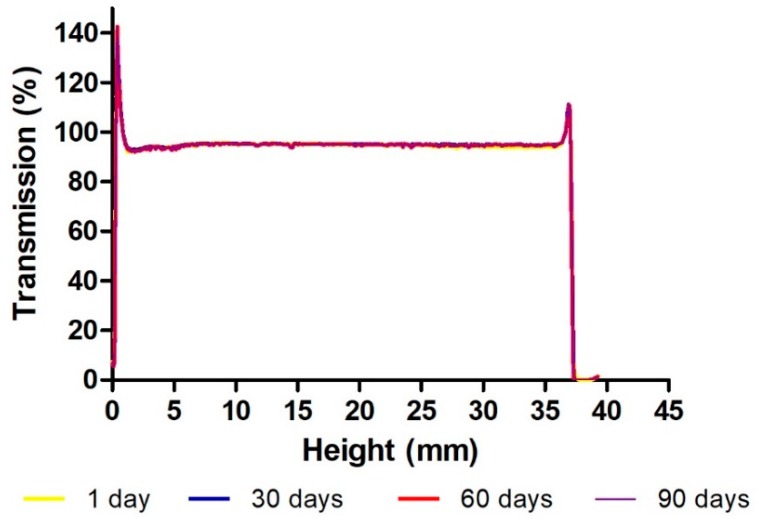
Transmission profile of AmB-gel obtained by Turbiscan Lab^®®^ over 90 days at 25 °C. The left side of the curve corresponds to the bottom of the vial, whereas the right side corresponds to the sample behavior on the top.

The pH value was suitable for application on the skin, ranging between 6.30 ± 0.05 and 6.70 ± 0.13 (*p* > 0.05) at 4 °C, and between 6.50 ± 0.13 and 6.67 ± 0.24 (*p* > 0.05) at 25 °C over a period of 90 days. The content of AmB present in the gel stored at 4 °C and 25 °C remained within the required margins of 90 to 100% during the first 90 days. With regards to the TurbiScan Lab^®®^ analysis, Figure 3 shows the transmission profile of AmB-gel at 25 °C over a period of 90 days. The lower part of the vial is represented on the left side of the graph and the upper part on the right side. The peaks in the lower part and in the upper part of the vial correspond to the meniscus produced by the sample from contact with the glass. No sedimentation, flocculation, or coalescence phenomena were observed.

### 3.3. Rheological Analysis

**Figure 4:**
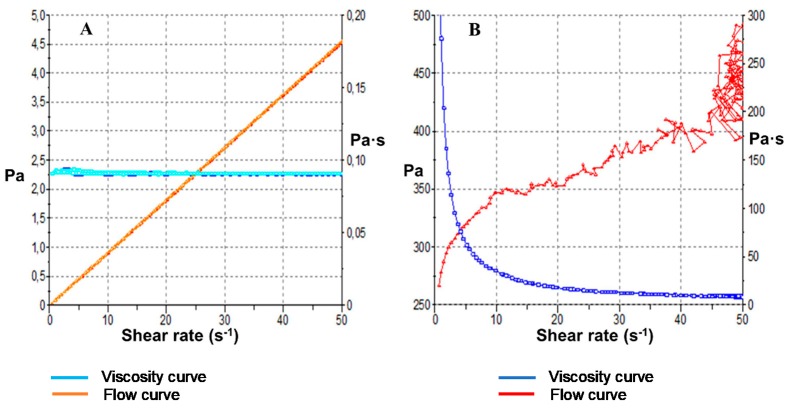
AmB-gel rheograms after 24 h: (**A**) Shear stress (Pa) and viscosity (Pa·s) curves at 4 °C and (**B**) Shear stress (Pa) and viscosity (Pa·s) curves at 32 °C.

Figure 4A reproduces the flow curves and the viscosity curves obtained from the rheological characterization of AmB-gel. At 4 °C, the gel was liquid and showed a Newtonian behavior (constant viscosity under shear rate increase) adjusted by the Newton model (*r*^2^ = 1) when under the programmed variations of shear rate. The viscosity at 4 °C was found to be 90.62 ± 7.112 × 10^−2^ mPa·s. Since AmB-gel transition is carried out as the temperature increases, at 32 °C a significantly sticky gel was formed which made it difficult to generate an adequate flow within the space between the conical plate. The rheogram (Figure 4B) showed a critical shear rate of ~10 s^−1^. When above this value, the sample could not flow freely under the experimental conditions. As a result, the layout became quite irregular and the results were inconclusive. However, the valid curve portion showed non-Newtonian behavior with properties of shear thinning (pseudoplastic behavior), thus adjusting the Cross model (*r*^2^ = 0.981). The punctual viscosity values at 10 s^−1^ and 32 °C determined from the ramp up period was ~3.8 Pa·s. The estimated viscosity of the gel at 32 °C and 50 s^−1^ was ~9.8 Pa·s.

### 3.4. Gelation Time and Spreadability

Gelation times of AmB-gel stored at 4 °C and 32 °C were 3 and 1.5 min, respectively. No differences in gelation times were evidenced between samples either with or without AmB. For AmB-gels stored for 3 months, the gelation times were even shorter at less than 1.5 min for 4 °C and 1 min 32 °C.

**Figure 5:**
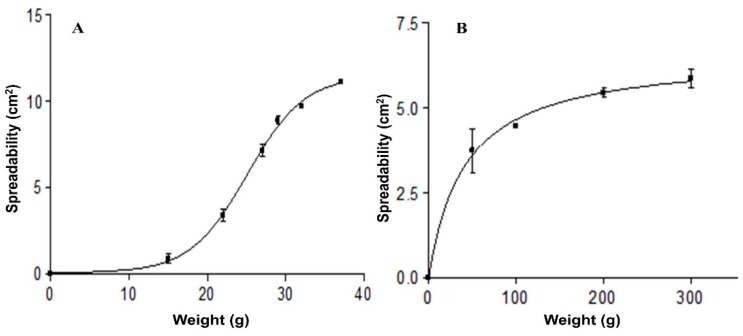
Spreadability charts of AmB-gel at different temperatures: (**A**) Spreadability curve at 4 °C; (**B**) Spreadability curve at 32 °C.

Figure 5 shows the results of spreadability test. The Boltzmann sigmoidal model for AmB-gel at 4 °C and hyperbola model for AmB-gel at 32 °C were the models that best fit the experimental data. At 4 °C, spreadability was greatly extended and with lower weight due to having lower viscosity at this temperature.

### 3.5. Release Studies

**Figure 6:**
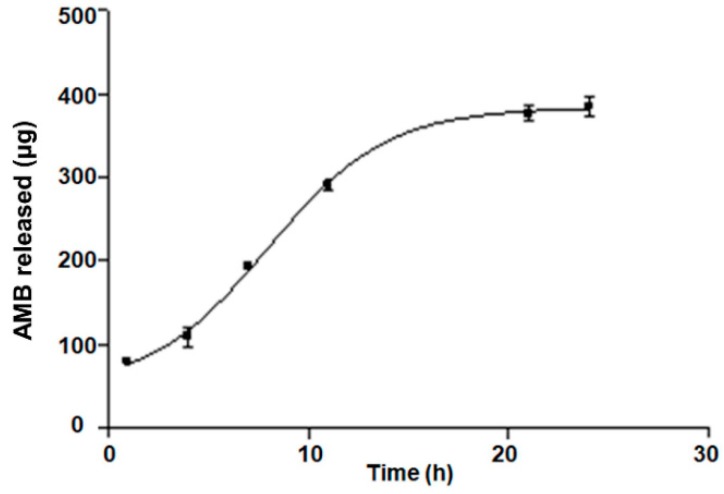
In vitro profile of AmB release from gel. The cumulative amount released was plotted against time. Data represent mean ± SD (*n* = 6).

Figure 6 shows the release profile of AmB from the gel, where a sustained release exhibiting S-shaped behavior can be observed. After 24 h, more than 90% of the drug was released. The optimal kinetic model was fitted to the Boltzmann sigmoidal according to the following equation with AIC of 30.05 and *r^2^* of 0.999:

(4) $$Y=Bottom+\left(Top\;-\;Bottom\right)/(1+\exp\left(\frac{V50-x}{Slope}\right)$$

where *Y* is the amount of drug released and *Top* and *bottom* are the initial and final values of drug release. *V50* is the time it takes to release half of the maximum amount susceptible for release and the slope of the curve indicates the steepness.

### 3.6. Ex Vivo Permeation Studies of Skin and Vaginal Mucosa

No amount of AmB from gel was detected in the receptor chamber in either skin or vaginal mucosa. Thus, neither flux nor permeation parameters could be calculated. The amount of AmB retained in the skin was 960.0 μg/g/cm^2^ and vaginal mucosa 737.52 μg/g/cm^2^.

### 3.7. Antifungal Efficacy

The values from the susceptibility test after 48 h are reported in Table 1 as MIC values against strains of *C. albicans*, *C. glabrata*, and *C. parapsilosis*. It can be observed that AmB-gel exhibited the lowest MIC values (0.09, 0.37 and 0.19 μg/mL) compared with Free-AmB.

### 3.8. Atomic Force Microscopy

**Figure 7:**
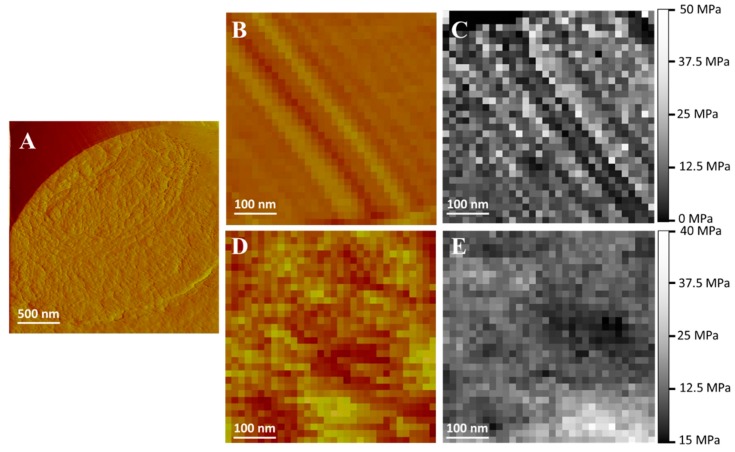
Effect of the Blank-gel on *C. albicans* cells. (**A**) Deflection image of a single yeast cell trapped in a pore. (**B**) Topographic map zoom image of the yeast cell before application of the Blank-gel. (**C**) Elasticity map zoom image of the yeast cell before application of the Blank-gel. (**D**) Topographic map zoom image of the cell yeast after application of the Blank-gel. (**E**) Elasticity map zoom image of the yeast cell after application of the blank-gel.

Figure 7 shows the effect of a Blank-gel on *C. albicans* cell. Figure 7A shows the deflection image of a single yeast cell trapped in a pore which appears as the reddish region at the top-left of the image while the cell surface shows an extended region covered by filamentous structures. Figure 7B,C depicts magnified images of topographic and elasticity maps of this region prior to the addition of blank-gel to a final concentration of 6.5% (*w*:*v*) as well as Figure 7D,E after addition. The surface was quite smooth before the addition of the Blank-gel and some filamentous structures could be observed and differentiated by its different Young’s modulus values with respect to the cell bulk structure (Figure 7B). After the addition of the blank-gel, the surface of the yeast cell became rougher and no filamentous structures could be observed any longer (Figure 7D). The elasticity map of the region (Figure 7E) evidences that it becomes more homogenous with the incorporation of polymer P407.

**Figure 8:**
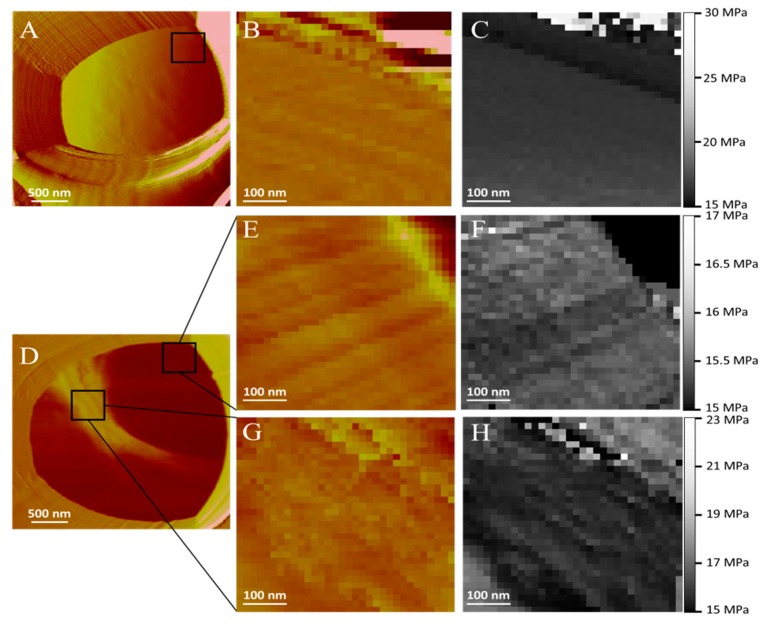
Effect of AmB-gel on *C. albicans* cells. (**A**) Deflection image of a single yeast cell trapped in a pore. (**B**) Topographic map zoom image of a single yeast cell trapped in a pore. (**C**) Elasticity map zoom image of a single yeast cell trapped in a pore. (**D**) A reddish region (low region) that covers almost the 80% of the cell surface and a yellowish arched band (high region) crossing the surface from top-left to center-right of the cell surface. (**E**) Topographic map zoom image of the low region. (**F**) Elasticity map zoom image of the low region. (**G**) Topographic map zoom image of the high region. (**H**) Elasticity map zoom image of the high region.

Figure 8 shows the effect of AmB-gel on *C. albicans* cells. Deflection, topographic and elasticity images (Figure 8A–C, respectively) of a single *C. albicans* yeast cell trapped in a pore are quite similar to those shown in Figure 6, although the filamentous structures are less evident in this case. The addition of AmB in the gel apparently altered the *C. albicans* cell. Figure 8D demonstrates that two different regions on cell surface can be clearly differentiated: (i) a reddish region (low region) that covers almost the 80% of the cell surface and (ii) a yellowish arched band (high region) crossing the surface from top-left to center-right of the cell surface protruding 40–80 nm over the reddish one. A close inspection of this yellowish band revealed a structure resembling a wrinkled sheet. Figure 8E,F shows the topographic and elasticity maps of the low region of Figure 8D. From these images, it is possible to recover the filamentous structures previously observed, although the differences in the Young’s modulus values between the filaments and the bulk structure are not overly distinctive. Figure 8G,H shows the topographic and elasticity maps of the high region of Figure 8D. From the images, it is possible to observe two differentiated regions: (i) one diagonal from top-left corner to the bottom-right corner of Figure 8H (darker region) and (ii) a second region formed from the higher Young’s values (lighter region) in Figure 8H occupying the bottom-left and top-right corners of the image with higher Young’s modulus values than those of the low region. Considering that the darker region in Figure 8H is the higher region of the topographic image (Figure 8G), it is possible to identify these “wrinkled sheets” as regions with low elasticity modulus, thus suggesting that material in these structures is not as packed as those with blank-gel.

**Figure 9:**
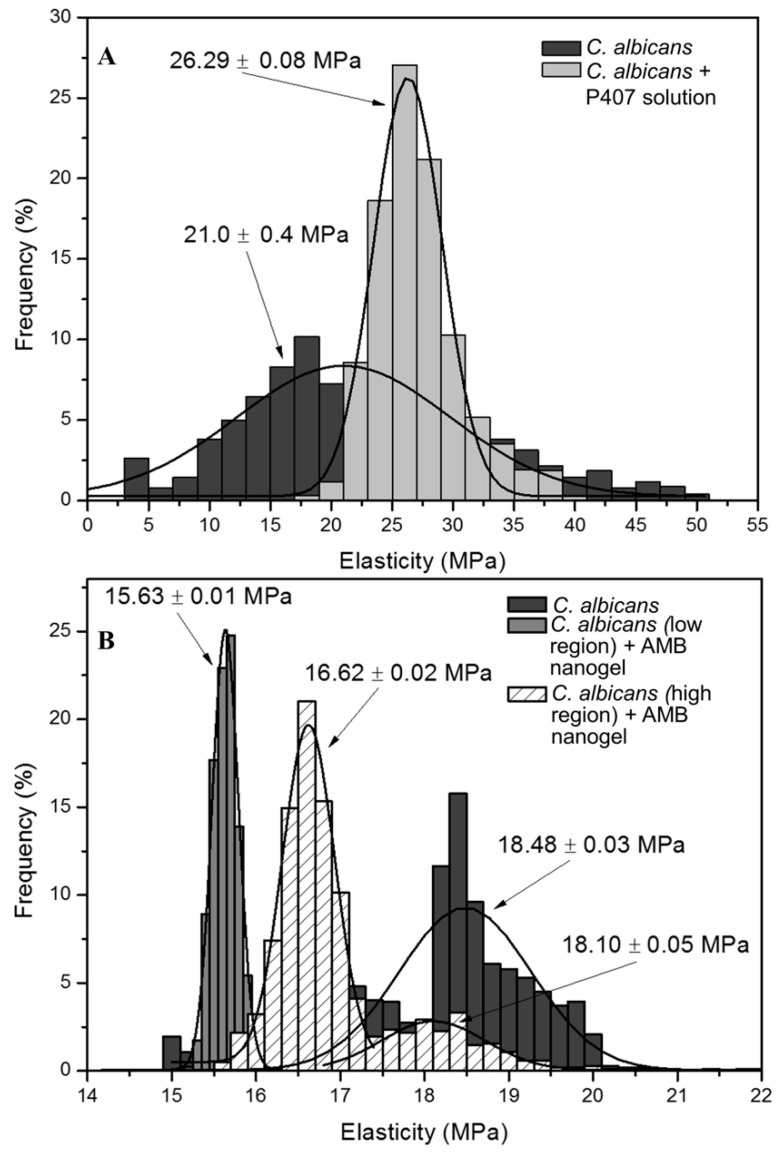
Individual Young’s modulus values as a function of its frequency on *C. albicans* cells. (**A**) Individual Young’s modulus values before and after the addition of Blank-gel. (**B**) Individual Young’s modulus values before and after the addition of the AmB-gel.

Conversely, Figure 9 shows the profiles of the elasticity values as a function of its frequency. Figure 9A represents the individual Young’s modulus values as a function of its frequency before and after the addition of blank-gel. Before the addition of blank-gel, the yeast cell showed a wide distribution of Young’s modulus values with a central value of 21.0 ± 0.4 MPa. After the addition of the blank-gel, the values distribution is narrower and centered at a higher value 26.29 × 0.08 MPa. Figure 9B shows a central peak around 18.48 MPa and a wide distribution function. The low region depicted in Figure 8D corresponds to a narrower peak with a Young’s modulus mean value that is shifted towards smaller values (15.63 MPa). On the contrary, the protruding structure resembling “wrinkled sheets” in Figure 8D shows a bimodal distribution, one close to the values of the blank *C. albicans* cell with a mean Young’s modulus value of 18.10 MPa and another shifted towards smaller values of 16.62 MPa.

### 3.9. In Vivo Tolerance Study by Evaluating Biomechanical Properties of Human Skin

**Figure 10:**
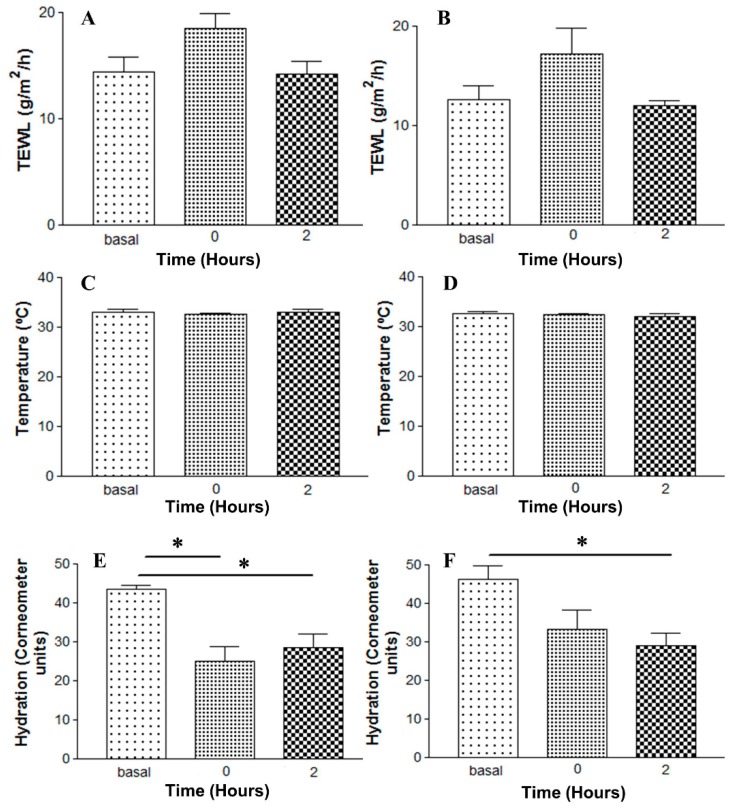
Biomechanical parameters evolution monitored before the application (basal), immediately after application (0 h) and 2 h after application. (**A**) TEWL values of blank-gel. (**B**) TEWL values of AmB-gel. (**C**) Skin temperature values of blank-gel. (**D**) Skin temperature values of AmB-gel. (**E**) SCH values of blank-gel. (**F**) SCH values of AmB-gel. * Statistically significant differences (*p* < 0.05).

The results of evaluated biomechanical parameters are depicted in Figure 10. No statistically significant changes of TEWL or skin temperature were observed between Blank-gel and AmB-gel or when compared with baseline measurements (*p* > 0.05). However, a significant decrease in SCH values was observed in the Blank-gel immediately after application (0 h) and at 2 h as well as in the AmB-gel after 2 h, with respect to the basal state (Figure 10E,F).

### 3.10. In Vivo Tolerance Study on Scarified Rabbit Skin by Draize Assay and Histological Analysis

The Draize test was carried out in order to evaluate the skin irritation potential of AmB-gel. After 48 h, the resulting primary irritation index value for Blank-gel and AmB-gel was 0.38 and 0.45, respectively. This result indicates that both blank-gel and AmB-gel are nonirritants.

**Figure 11:**
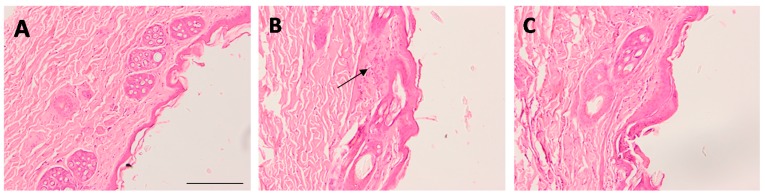
Optical microscopic images of skin. Blank-gel (**A**), skin-scarified control group (**B**), and AmB-gel (**C**). Hematoxylin and eosin stains nuclei blue/black while keratin and cytoplasm are stained red. Scale bar = 200 μm.

Regarding the histological evaluation, micrographs revealed that scarification caused histological alterations and the presence of nonspecific inflammatory cells in the skin (Figure 11B) while the topical application of Blank-gel (Figure 11A) and AmB-gel (Figure 11C) notably repaired these alterations, resulting in a less pronounced inflammatory process than that of the control group (Figure 11B).

## 4. Discussion

P127 gels are used as the vanguard of drug delivery systems development due to the improved therapeutic efficacy and adherence to the recipient [24]. They have been used as vehicles for various routes of administration of drugs, including oral and topical [25], intranasal [18,26], vaginal and rectal [27], ocular [28], and parenteral [29]. In this study, AmB-gel was developed with 5% of DMSO as an optimal solubilizing agent and 25% of P127 to confer thermoreversible character. For the physicochemical characterization, SEM images were obtained, thus confirming that AmB-gel has a dense and well-oriented tubular appearance along with reticular networks. This type of structure has been reported in other studies [30,31], therefore supporting the postulation that a porous structure could help in the controlled release of drug.

The swelling of AmB-gel is a parameter dependent on the critical micelle concentration (CMC), given that P127-gels show two forms: gel form at 25 °C and liquid form at 4 °C, with liquid form showing thermoreversible behavior. Once the AmB-gel is poured into the swelling medium (PBS), a concentration of P407 decreases rapidly below CMC, thus producing swelling and solubilization in ~19 min. In the present study, 25% of P127 was used and at this concentration the SR followed a first-order kinetic model. Additionally, the degradation process was complete at 20 min, which is a typically rapid rate of degradation for these kinds of systems, as previous studies have also found [32]. On the other hand, the AmB-gel percentage of P was about 82.01 ± 0.5% corroborated with the SEM image which showed a high porosity structure.

The pH value of skin formulations is an important factor to consider in order to avoid skin irritation, particularly when under mycosis infections. AmB-gel showed pH levels to be slightly acidic, therefore biocompatibility with the natural acidity of the skin assures suitability for skin application [30]. What is more is that the AmB content in the gel was maintained between 90 and 100% over a span of 3 months while the superimposed graphs of transmission (Figure 3) showed variations below 10%, which indicates that the product will remain physically stable for a minimum of three months [33,34,35].

As expected, the rheological characteristics of AmB-gel were temperature-dependent (Figure 4). It is vital to evaluate rheological characterization because rheology can modulate biopharmaceutical properties such as release rates in addition to the widespread application on affected areas [36]. The viscosity at 4 °C was fairly low at ~90 mPa·s, which is an important value at the time of packaging because greater liquescence can lead to optimal forms of delivery, such as spray or roll-on applications, which help prevent the spread of mycotic infections. Once in contact with the affected skin at 32 °C, the solubility of the PPO chains decreases, subsequently forming micelles and assuming hexagonal and/or cubic structures, thus drastically increasing viscosity. The estimated viscosity of the AmB-gel at 32 °C and 50 s^−1^ was ~9.8 Pa·s, which is nearly 100 times higher than the viscosity at 4 °C. This thermal gelation of P407 can form and sustain a drug depot in this area and subsequently increase the contact time, which can then produce desirable drug release characteristics and prolong pharmacological action [37]. The thermal gelation process did not seem to be affected by the presence of AMB. All the results suggested that gel state would be reached after application on the skin and also gain viscosity instantaneously to increase residence time of the formulation on the application area [13].

The data obtained from release studies demonstrated that the AmB-gel provided a sustained release for approximately 24 h. Model fitting showed that release mechanism followed a sigmoidal model. This faster release could be attributed to the highly porous structure and the rapid degradation of P407-gel. Moreover, this mathematical model has been associated with these types of gels due to the fact that they are used to simulate the transport and reaction of fluid in porous media [38].

Ex vivo permeation studies revealed that AmB did not permeate through human skin nor porcine vaginal mucosa. Similar results were reported in previous studies in which no AmB from a nanoemulsion was detected in the ex vivo permeation study using human skin or pig’s ear skin [12,39], which might be explained by the great molecular weight (926 D) and high hydrophobicity of AmB, effectively limiting its ability to pass through the aqueous structure of the vaginal mucosa and the hydrophilic barrier of the dermis. However, it was observed that AmB had high retention in the skin and vaginal mucosa with values of 960 μg/g/cm^2^ and 737 μg/g/cm^2^, respectively. This result confirms that AmB succeeded in crossing the SC and was distributed to both the epidermis and dermis. Furthermore, it was able to permeate into the vaginal mucosa without reaching the systemic circulation, which was confirmed with the high amount of drug retained in the tissue and is indicative of the formulation favoring a local effect on the target area with no side effects. This high drug retention capacity in both tissues could be due to the presence of P407 in the formulation, since the chemical structure of this polymer allows it to act as a surfactant that enhances the diffusion ability of drug, resulting in increased drug concentrations in the tissue layer that leads to efficiently provide a local effect [18]. These results suggest that the AmB in this formulation could be successfully implemented in order to achieve a local effect on the skin or vaginal mucosa without adverse systemic effects.

The antifungal action of AmB-gel was clearly observed (Table 1) with a MIC value lower than Free-AmB in all tested Candida species and thus was highly effective—essentially confirming the adequacy of the formulation due to its improved effectiveness against fungus and yeast infections, which is likely due to the synergistic effects reported between the formulations of poloxamers and antifungal agents [40]. On the other hand, Blank-gel did not have any effect against Candida strains and produced values consistent with those reported in previous studies [12]. These results were subsequently observed by AFM images (Figure 7 and Figure 8) which evidenced the alterations on the surface of Candida yeast cells that were induced by AmB-gel. In an equivocal manner, the mean values of Young’s modulus (Figure 9) demonstrate the destructuring effect of AmB-gel on the outer membrane of the yeast cell.

The tolerance of the formulation was studied by evaluating the biomechanical properties of skin, since parameters such as TEWL and SCH are important indicators of skin integrity. Thus, any changes in these parameters could be directly related to an alteration of the functionality of the skin barrier [41]. From the results of this noninvasive in vivo method, no statistically significant differences were observed in temperature or TEWL values of volunteers after treatment with AmB-gel or with blank-gel when compared with baseline measurements (Figure 10), whereas SCH values showed a significant reduction in both AmB-gel and blank-gel with respect to the basal state. This decrease in the hydration levels in the area of application of the final product could be due to the ability of the gel to absorb moisture produced by the sweat glands. When the formulation containing P407 dries on the surface of the skin, it forms a film that captures moisture and does so in a state of relative equilibrium with ambient humidity. This effect would be especially suitable in skin candidiasis because it could prevent the proliferation of microorganisms around the affected area [42,43]. Moreover, when comparing the values of the biomechanical parameters evaluated for AmB-gel and blank-gel, the incorporation of the AmB does not significantly modify the properties of the gel. Furthermore, none of the volunteers exhibited side effects of burning or itching after the application. Therefore, the AmB-gel did not alter the biophysical properties of the skin of volunteers throughout the course of the study.

Finally, the tolerability of the formulation was evaluated by in vivo model using scarified rabbit skin in order to simulate the damage of the skin barrier caused by the fungal infection. The irritation index values for AmB-gel and Blank-gel were less than 0.5 and can therefore be classified as nonirritant formulations. This result was consistent with histological analysis, which showed that both formulations did not cause any alteration of the skin architecture but, on the contrary, they actually repaired the ulcerated skin. According to this finding the ability of P407 to reduce wounds in burn patients while simultaneously stimulating the proliferation of collagen fibers, consequently increasing scarring and generating new tissue, have all been reported in previous studies [44]. In conjunction, these effects might favor the healing and alleviation of wounds caused by Candida. In conclusion, AmB-gel represents a promising therapeutic option to treat these types of infections via dermal application.

## 5. Conclusions

The focus of the present study was on the incorporation of AmB in a copolymer P407-based gel designed for application on both the skin and vaginal mucosa, specifically for the treatment of candidiasis. P407 has shown great potential as a support system in the administration of hydrophilic and hydrophobic drugs, as is the case of AmB. P407 is suitable due to its thermoreversible capacity, low toxicity, and permeation-enhancing properties. During the experimental phase, AmB-gel remained stable for at least 3 months, thereby demonstrating pH values suitable for both skin and vaginal application. Due to its low viscosity at predetermined temperatures and its porous structure which facilitates rapid drug release, AmB-gel can also be used in aerosol form in order to avoid contact with areas infected by the fungus and thus prevent the spread of infection to other anatomical sites while minimizing the risk of a subsequent superinfection. No drug was quantified in the receptor compartments of the Franz cells, which indicates nonabsorption of AmB and therefore avoids adverse systemic effects. None of volunteers exhibited burning or itching after application and the histological images of rabbit skin displayed high tolerability of the formulation. Furthermore, AmB-gel did not alter the biophysical properties of skin, thus confirming adequate safety as topical agents. According to the values of the amount of drug retained in the skin and vaginal mucosa, along with the MIC values and the images obtained by AFM, it can be concluded that AmB-gel provides a satisfactory antifungal effect across various Candida species.

## Figures and Tables

**Table 1:** MIC against different cultures of *Candida* spp., Free-AmB, AmB-gel, and Blank-gel after incubation at 30 °C for 48 h (*n* = 3).

Tested Species	Origin	MIC (µg/mL)		
		Free-AmB	AmB-gel	Blank-gel
*C. albicans*	ATCC 10,231	0.15	0.09	>250.000
*C. glabrata*	ATCC 66,032	0.60	0.37	>250.000
*C. parapsilosis*	ATCC 22,019	0.30	0.19	>250.000
